# Resolution of Sleepwalking Behavior after Initiation of Sodium Oxybate in a Patient with Narcolepsy Type 2

**DOI:** 10.7759/cureus.2662

**Published:** 2018-05-21

**Authors:** Sean Hesselbacher, Amir Sharafkhaneh

**Affiliations:** 1 Medicine, Hampton Veterans Affairs Medical Center, Hampton, VA, USA; 2 Medicine, Baylor College of Medicine; Michael E. Debakey Veterans Affairs Medical Center

**Keywords:** narcolepsy, sleepwalking

## Abstract

A 44-year-old male veteran with long-standing excessive daytime sleepiness was diagnosed with Narcolepsy Type 2. The patient was unable to tolerate effective doses of methylphenidate, due to mood disturbances, or modafinil, due to adverse gastrointestinal effects. Although the patient also reported an ongoing history of sleepwalking with potentially injurious behaviors, a cautious trial of sodium oxybate was initiated. The combination of sodium oxybate and low dose methylphenidate resulted in significant improvement in patient-reported subjective daytime sleepiness. Additionally, self-report of the sleepwalking behaviors markedly improved. This case shows that patients with narcolepsy and sleepwalking may still safely benefit from a cautious trial of sodium oxybate.

## Introduction

Narcolepsy is a rare group of hypersomnia disorders which are estimated to occur in 25-50 per 100,000 people [1]. In the most recent version of the International Classification of Sleep Disorders, narcolepsy is classified based on the presence (“Narcolepsy Type 1”) or absence (“Narcolepsy Type 2”) of cataplexy [2]. The primary symptom of both types of narcolepsy is excessive daytime sleepiness, which is required for diagnosis; other ancillary symptoms include cataplexy (in Narcolepsy Type 1), sleep paralysis, hypnogogic or hypnopompic hallucinations, and automatic behaviors. These symptoms, especially excessive daytime sleepiness, can be disabling in many cases and present public health hazards, if not adequately treated. Sleep testing is required to diagnose either type of narcolepsy: in the appropriate clinical context, the findings of short sleep latency and two or more periods of Rapid Eye Movement (REM) sleep at sleep onset (sleep-onset REM periods) on multiple sleep latency test (MSLT) are consistent with narcolepsy; classification is typically based on patient-reported symptoms.

Both types of narcolepsy are often managed with wake-promoting agents, such as stimulants or modafinil. Sodium oxybate is a non-stimulant medication that reduces symptoms of cataplexy and excessive daytime sleepiness in narcoleptic patients. Many patients with either type of narcolepsy have other comorbid sleep disorders, which may complicate treatment efforts. For example, sodium oxybate may cause or exacerbate non-REM parasomnia behavior or sleep-disordered breathing. REM sleep behavior disorder and non-REM parasomnias have been reported in patients with narcolepsy [3, 4], and obstructive sleep apnea is common in these patients.

## Case presentation

A 44-year-old male veteran was seen in follow-up of long-standing daytime sleepiness, characterized by sudden onsets of sleep, even while standing up, and lost time of up to 30 minutes. He denied cataplexy, hypnogogic hallucinations, or sleep paralysis. Initial evaluation was performed in another state in 1993; the report from the sleep studies done at that time indicated “32 awakenings, 2 apnea/hypopnea spells, mean sleep onset 5.9 min x 5 naps (no sleep on one nap), observed to enter REM sleep in at least two naps and possible four of five naps.”

He relocated and subsequently presented for re-evaluation to our sleep disorders center in 2004, with persistent complaints of excessive daytime sleepiness, not on specific treatment. He also reported ongoing sleepwalking episodes: some episodes involved walking on the roof of a chemical plant where he was working or smoking a cigarette on his front porch. An overnight polysomnogram (PSG) and MSLT were repeated. There was no significant sleep-disordered breathing, or abnormal increase in muscle tone or unusual activity during REM or non-REM sleep. During the MSLT, sleep occurred on all four nap opportunities, the mean sleep latency was 10 minutes and there were no sleep onset REM periods. Initial treatments included stimulant medications and extension of the nocturnal sleep period. Methylphenidate at 20 mg per day was ineffective in maintaining wakefulness; doses up to 60 mg per day resulted in unacceptable irritability and anger. A trial of modafinil was not tolerated due to adverse gastrointestinal effects.

**Figure 1:**
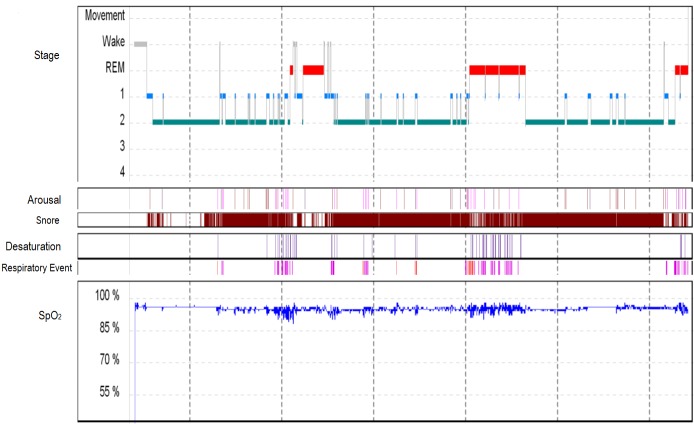
Graphical representation of the overnight polysomnogram performed in 2008. SpO_2_: Peripheral oxygen saturation; REM: Rapid Eye Movement.

**Figure 2:**
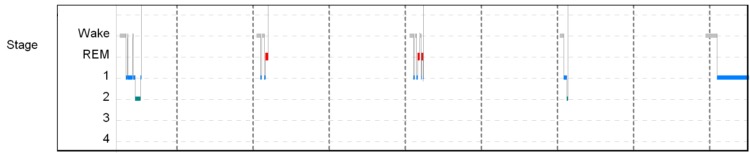
Graphical representation of the multiple sleep latency test performed in 2008. REM: Rapid Eye Movement.

A repeat PSG (Figure 1) and MSLT (Figure 2) were performed in 2008. On this occasion, the PSG showed excellent sleep efficiency (96.5%), normal REM latency (93.5 minutes), REM sleep comprised 16.8% of the total sleep time, and slow wave sleep and periodic limb movements of sleep were absent. Mild obstructive sleep apnea was present, with an apnea-hypopnea index of 9.1 events per hour of sleep and low oxygen saturation of 89%. During the subsequent MSLT, sleep occurred on all five nap opportunities, the mean sleep latency was 2.6 minutes, and REM sleep occurred on naps two and three.

The patient did not tolerate a trial of continuous positive airway pressure (CPAP) due to nasal obstruction from a prior nasal fracture. He again did not tolerate effective doses of traditional stimulant medications. For treatment of narcolepsy without cataplexy, sodium oxybate was initiated, 2.25 grams at bedtime and repeated four hours later, in addition to methylphenidate at 20 mg per day. On follow-up, the patient reported consolidated sleep, vivid dreams, and improved daytime sleepiness; he additionally reported a substantial decrease in the frequency of sleepwalking episodes and no related injuries.

## Discussion

This patient presented a therapeutically challenging case of Narcolepsy Type 2, for which the typical treatments (wake-promoting agents) were not well-tolerated. Mild obstructive sleep apnea was identified on the most recent overnight PSG, though it was unlikely to be a major contributor to this patient’s daytime sleepiness, as the sleepiness predated the new finding of sleep-disordered breathing.

Sodium oxybate is the sodium salt of gamma-hydroxybutyrate, an endogenous compound and metabolite of the neurotransmitter gamma-aminobutyric acid (GABA) [5, 6]. It is hypothesized that the therapeutic effects of sodium oxybate are mediated through GABA(B) actions at noradrenergic and dopaminergic neurons, as well as at thalamocortical neurons, though there may also be some modulation of GABA(A) receptors. Effects on sleep architecture include overall reduction in nocturnal sleep disruption, increase in duration and intensity of slow wave sleep, decrease in light sleep, decrease in nocturnal awakenings, and decrease in total REM sleep [7]. Clinical effects include decreased daytime sleepiness, as well as resolution of cataplexy, sleep paralysis, and sleep-onset hallucinations [8]. In the United States, it is indicated for the treatment of daytime sleepiness in patients with narcolepsy [5]. However, the presence of potentially hazardous sleepwalking made the use of sodium oxybate, which has been reported to worsen sleepwalking disorder [5], particularly problematic. We opted for a cautious trial of sodium oxybate in this case. Not only did the daytime sleepiness improve, as expected, but the patient-reported sleepwalking behaviors markedly improved as well.

Likely explanations for the improvement in sleepwalking behaviors include consolidation of sleep by sodium oxybate, myorelaxant properties of sodium oxybate, interruption in the sleep pathways leading to sleepwalking, or mischaracterization of the parasomnia. Sleepwalking is a disorder of partial arousal from non-REM sleep or during transitions, coupled with mechanisms to maintain sleep [9]; therefore, reduction of arousals by sodium oxybate may effectively eliminate sleepwalking and related parasomnias. GABA(B) agonists effectively relax skeletal muscle, which may prevent the partial arousals from transitioning to sleepwalking [6]. The recommended dosing structure of sodium oxybate requires a dose immediately before bedtime, followed by a second dose four hours later; patients often wake themselves for the second dose [5]. It is possible that this awakening interrupts the slow-wave sleep cycle that would otherwise lead to sleepwalking, similar to strategies whereby parents awaken children prior to the time of night that recurrent night terrors or sleepwalking typically occur. Alternatively, the patient may have been afflicted by REM sleep behavior disorder (RBD) rather than sleepwalking. The patient’s history was most consistent with sleepwalking, including the types of behaviors and timing during the night; there was also no loss of REM atonia noted on the PSG. RBD has been reported to be lessened in some patients taking sodium oxybate, which is contrary to the reported consequences for sleepwalking disorder [10]. Less likely explanations for the sequence of events, that must still be considered, include resolution of sleepwalking as the natural history of the disorder, and malingering.

## Conclusions

Our patient obtained substantial benefit from sodium oxybate with regards to both disabling daytime sleepiness and potentially dangerous sleepwalking behavior. Sodium oxybate has long been associated with new-onset sleepwalking or exacerbation of pre-existing sleepwalking, though this case represents an exception. We have reviewed some of the mechanism by which the improvement in parasomnia behavior may occur. With careful monitoring, sodium oxybate may still be considered in patients with comorbid narcolepsy and sleepwalking disorder.
